# Identification of shared gene signatures in major depressive disorder and triple-negative breast cancer

**DOI:** 10.1186/s12888-024-05795-z

**Published:** 2024-05-16

**Authors:** Hua Xie, Chenxiang Ding, Qianwen Li, Wei Sheng, Jie Xu, Renjian Feng, Huaidong Cheng

**Affiliations:** 1 Department of Oncology, The Second Affiliated Hospital of Anhui Medical University, Furong Road 678, Shushan District, Hefei, 230601 Anhui China; 2 Xuancheng People’s Hospital, Affiliated Xuancheng Hospital of Wannan Medical College, Dabatang Road 51, Xuanzhou District, Xuancheng, Anhui 242000 China; 3 Bengbu Medical College, Donghaida Road 2600, Longzihu District, Bengbu, Anhui 233030 China; 4 Mental Health center of Xuancheng City, Changqiaocun Jinba Road, Economic and Technological Development Zone, Xuancheng, Anhui 242000 China; 5 Department of Oncology, Shenzhen Hospital of Southern Medical University, Xinhu Road 1333, Bao’an District, Shenzhen, Guangdong 518000 China

**Keywords:** Major depressive disorder, Triple-negative breast cancer, WGCNA, Hub DEG

## Abstract

**Background:**

Patients with major depressive disorder (MDD) have an increased risk of breast cancer (BC), implying that these two diseases share similar pathological mechanisms. This study aimed to identify the key pathogenic genes that lead to the occurrence of both triple-negative breast cancer (TNBC) and MDD.

**Methods:**

Public datasets GSE65194 and GSE98793 were analyzed to identify differentially expressed genes (DEGs) shared by both datasets. A protein-protein interaction (PPI) network was constructed using STRING and Cytoscape to identify key PPI genes using cytoHubba. Hub DEGs were obtained from the intersection of hub genes from a PPI network with genes in the disease associated modules of the Weighed Gene Co-expression Network Analysis (WGCNA). Independent datasets (TCGA and GSE76826) and RT-qPCR validated hub gene expression.

**Results:**

A total of 113 overlapping DEGs were identified between TNBC and MDD. The PPI network was constructed, and 35 hub DEGs were identified. Through WGCNA, the blue, brown, and turquoise modules were recognized as highly correlated with TNBC, while the brown, turquoise, and yellow modules were similarly correlated with MDD. Notably, *G3BP1*, *MAF*, *NCEH1*, and *TMEM45A* emerged as hub DEGs as they appeared both in modules and PPI hub DEGs. Within the GSE65194 and GSE98793 datasets, *G3BP1* and *MAF* exhibited a significant downregulation in TNBC and MDD groups compared to the control, whereas *NCEH1* and *TMEM45A* demonstrated a significant upregulation. These findings were further substantiated by TCGA and GSE76826, as well as through RT-qPCR validation.

**Conclusions:**

This study identified *G3BP1, MAF, NCEH1* and *TMEM45A* as key pathological genes in both TNBC and MDD.

## Introduction

Breast cancer (BC) is one of the most common and fatal malignancies worldwide [1]. Considering the long latency and young age of onset, it is important to identify individuals susceptible to BC. Various genetic and environmental factors have been identified to lead to elevated risk of BC. Emerging evidence suggests that patients with major depressive disorder (MDD) have an increased risk of BC [2], and that genetic predisposition of MDD was causally associated with BC risk [3]. This implies that certain genetic and molecular pathogenic factors of MDD may also contribute to the development of BC. In 2011, the St. Gallen Expert Consensus divided BC into four subtypes [e.g., luminal A (expressing the oestrogen receptor (ER+), luminal B (ER+), HER2+ (without ER expression (ER-), and triple-negative breast cancer (TNBC, ER-)] [1, 4], of which TNBC was the most aggressive subtype and had the worst prognosis [5]. Recent research has shown that MDD is a risk factor for ER-negative breast cancer [3]. Thus, we hypothesized that MDD and TNBC share common pathological mechanisms.

MDD is caused by both genetic and environmental factors, and its etiology involves multiple organs, including the endocrine, nervous, and immune systems [6–8]. Although emerging genetic and epidemiological evidence suggests that MDD and BC may have similar etiological mechanisms, the specific pathogenic pathways and molecules that they share have not yet been clearly elucidated. In this study, we identified differentially expressed genes (DEGs) that are commonly associated with both MDD and TNBC by analyzing high-throughput sequencing data from public databases, selected hub DEGs, and further validated their biological importance in functional assays.

## Materials and methods

### Sample and dataset collection

Expression data for TNBC and MDD were obtained from the Gene Expression Omnibus (GEO) database (https://www.ncbi.nlm.nih.gov/geo/). The GSE65194 dataset [9–11] was analyzed using the GPL570 [HG-U133_Plus_2] Affymetrix Human Genome U133 Plus 2.0 array consisting of 55 TNBC samples and 11 control samples. The GSE98793 dataset [12] contains samples with sex information labeled “Female” and was analyzed in 96 MDD samples and 48 controls using the GPL570 [HG-U133 _ Plus _ 2] Affymetrix Human Genome U133 Plus 2.0 array.

Blood samples from five patients with MDD, five matched healthy individuals, tumor tissue samples, and normal adjacent tissue samples from five patients with BC were collected during the time period between January 2022 and March 2023.

### Identification of common differentially expressed genes (DEGs)

The “limma” package [13] (v3.34.7, https://bioconductor.org/packages/release/bioc/html/limma.html) in the R software (v4.3.1) was employed to identify DEGs in the TNBC dataset GSE65194 and the MDD dataset GSE98793. Genes with a false discovery rate (FDR) < 0.05, and |Log2FoldChange| > 0.5 were considered DEGs. TNBC and MDD DEGs intersected, and genes that were significantly upregulated or downregulated in both sets were selected as common DEGs. Gene Ontology (GO) and Kyoto Encyclopedia of Genes and Genomes (KEGG) analyses of these common DEGs were conducted in the Database for Annotation, Visualization and Integrated Discovery (DAVID) database (v.6.8, https://david.ncifcrf.gov/) [14, 15], and FDR < 0.05 was considered significantly enriched. Functional annotations in the GO analyses included biological process (BP), cell component (CC), and molecular function (MF).

### Protein-protein interaction (PPI) network construction and identification of hub genes

An interaction network of the common DEGs was constructed using STRING (v. 11.0, http://string-db.org/), with the minimum required interaction score set to 0.4, and the results were visualized using the online tool Cytoscape (v.3.9.0, http://www.cytoscape.org/) [16]. The CytoHubba plug-in of Cytoscape [17] was employed to identify hub genes, and four topological analysis methods were applied: Maximal Clique Centrality (MCC), Maximum Neighborhood Component (MNC), degree, and Edge Percolated Component (EPC). The top 50 genes resulting from each method were intersected, and the genes present in all four sets were chosen as hub genes.

### Weighted gene co-expression network analysis (WGCNA) and selection of hub DEGs

The R package WGCNA (v.1.72-1, https://cran.r-project.org/web/packages/WGCNA/index.html) [18] was applied to perform weighted gene co-expression network analysis (WGCNA). We selected a height cut corresponding to a correlation of 0.99, and the minimum modulus was set to 100. WGCNA modules were significantly positively correlated with both TNBC and MDD (|Pearson correlation coefficient (PCC)| > 0.3), and genes in these modules intersected with previously selected hub genes. Genes present in both sets were identified as hub DEGs for the two diseases. Based on the expression levels of the hub DEGs, principal component analysis (PCA) was conducted to assess whether their expression was disease-specific. The expression levels of hub DEGs were extracted from all four datasets, and their expression levels in disease samples were compared with those in control samples in each of the four datasets.

### Identification of the disease-associated “miRNA-hub DEG” interactions and functional annotation of hub DEGs

TNBC-associated miRNAs and MDD-associated miRNAs were downloaded from the Human MicroRNA Disease Database (HMDD) v4.0 (http://www.cuilab.cn/hmdd) [19] and intersected to obtain the miRNAs that were correlated with both diseases. MiRNAs potentially regulating hub DEGs were predicted using the miRWalk v3.0, database (http://129.206.7.150/) [20]. KOBAS v3.0 database (http://kobas.cbi.pku.edu.cn/) and was used to annotate the KEGG pathways of the hub DEGs. The results were visualized in a Sankey diagram drawn using the R package ggplot2 (v3.4.4; https://cran.r-project.org/web/packages/ggplot2/index.html).

### Validation of hub DEGs in independent external datasets and using real-time quantitative reverse transcription-polymerase chain reaction (RT-qPCR)

To enhance the robustness of our findings, we validated the expression levels of hub DEGs using the TCGA and GSE76826 datasets, complemented by RT-qPCR analysis. Expression data for breast cancer were retrieved from the TCGA database, encompassing 158 TNBC samples and 113 normal control samples, using the Illumina HiSeq 2000 RNA Sequencing platform employed for detection. The GSE76826 dataset was generated using the GPL17077 Agilent-039494 SurePrint G3 Human GE v2 8 × 60 K Microarray 039381 (probe name version), including samples annotated as “Female,” comprising 11 MDD samples and 7 controls.

The *“TransZol* Up” reagent was used to isolate total RNA from blood or tissue samples (TransGen Biotech Inc., Beijing, China, ET111-01). The SYBR Green RT-PCR assays were performed following the manufacturer’s instructions for “First-Strand cDNA Synthesis SuperMix for qPCR” (TransGen Biotech Inc, AU341-02) and “PerfectStart® Green qPCR SuperMix” (TransGen Biotech Inc, AQ601-02). Reactions were run on a LongGene Q2000B system (LongGene Inc., Hangzhou, Zhejiang, China). Three independent experiments were conducted for statistical significance, and all assays were performed in triplicate. The 2^−ΔΔCt^ method was applied for the relative quantification of gene expression levels. The primer sequences are listed in Table 1.

**Table 1:** Primers of the real-time reverse transcription-polymerase chain reaction

Gene	Forward primer sequence (5’-3’)	Reverse primer sequence (5’-3’)
GAPDH	TGACAACTTTGGTATCGTGGAAGG	AGGCAGGGATGATGTTCTGGAGAG
G3BP1	AGAGGAGCCTGTTGCTGAAC	CTGCAGGTGCTGGAGAAGAA
MAF	cctggccatggaatatgttaat	agccggtcatccagtagtagtc
NCEH1	ggccacaaagtatttcctgaag	gggtgttcacattttgctgata
TMEM45A	accaatgactcagaagggaaaa	ttttggaaccaagatagcaggt

### Statistical analyses

All bioinformatics analyses were performed using the R software (v.4.3.1). RT-qPCR data were analyzed using the Student’s t-test in GraphPad Prism software (v.9.5.1). *p* < 0.05 was considered statistically significant.

## Results

### Identification of common DEGs and functional enrichment analyses

**Fig. 1:**
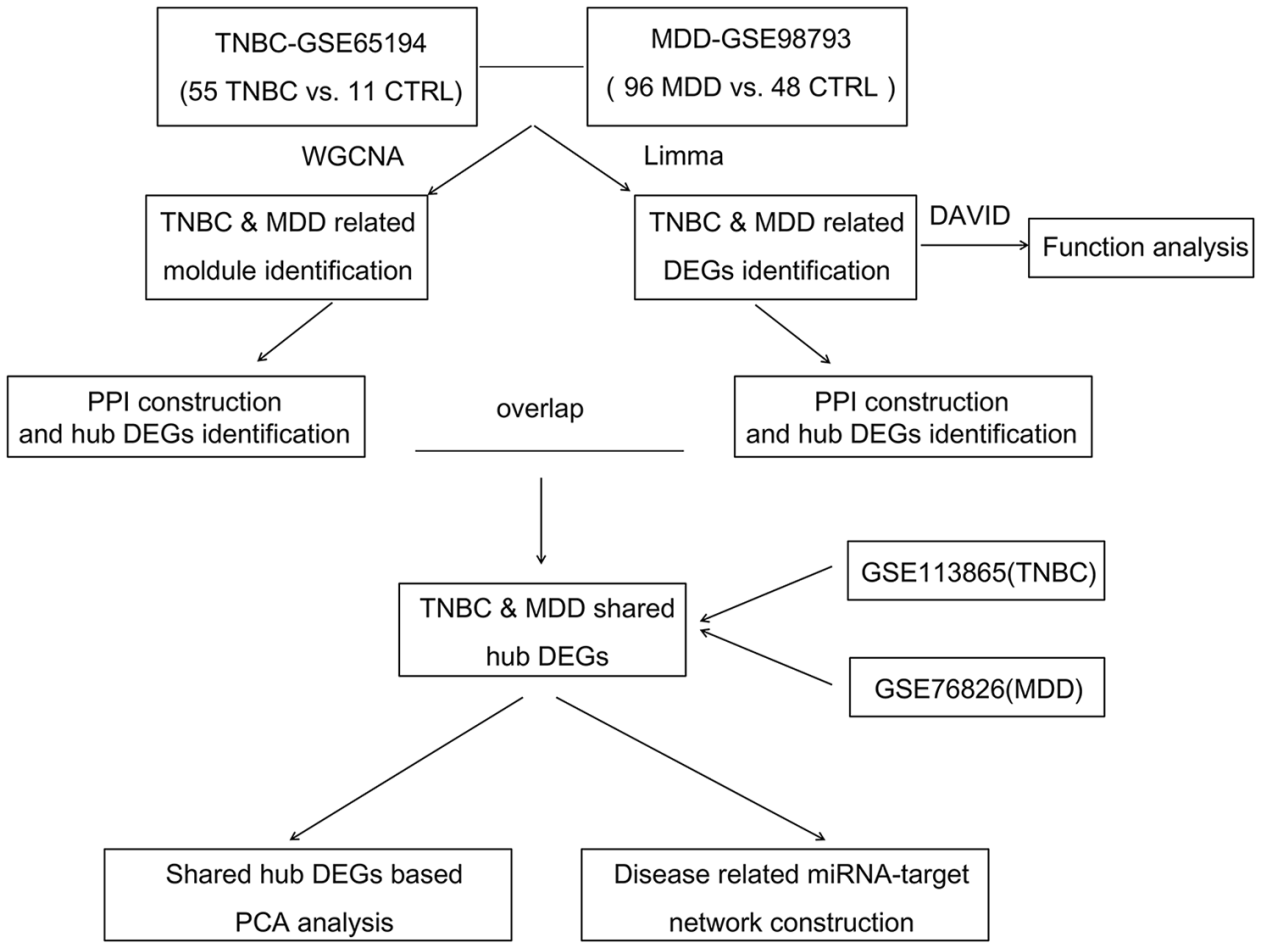
Flow diagram of the study

**Fig. 2:**
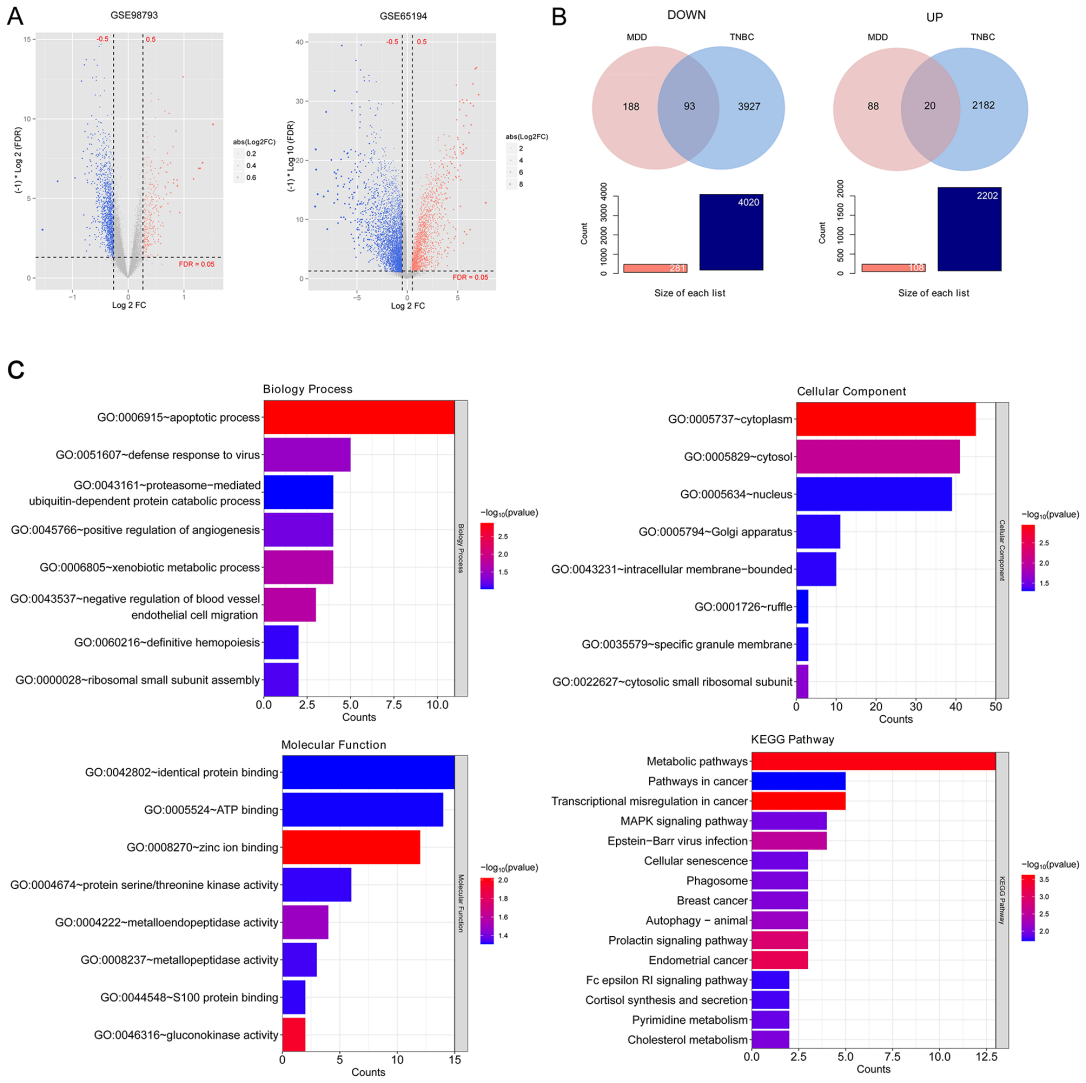
Identification of common differentially expressed genes (DEGs) and hub genes.(**A**) Volcano plots showing DEGs. Red and blue dots indicate significantly up-regulated and down-regulated genes in the disease group compared to the control. (**B**) Venn diagrams displaying the common DEGs that were down-regulated in both diseases (left) and up-regulated in both diseases (right). (**C**) Enrichment analyses of the common DEGs. All the identified Gene Ontology terms and the top 15 enriched Kyoto Encyclopedia of Genes and Genomes pathways were shown

Figure 1 shows the flow diagram of this study. A total of 2,202 and 108 genes were significantly upregulated in TNBC and MDD samples, respectively. Additionally, 4,020 and 281 genes were significantly downregulated in TNBC and MDD samples, respectively, as shown in the volcano plots (Fig. 2A). Twenty genes were significantly upregulated in both TNBC and MDD samples, and 93 genes were significantly downregulated in both TNBC and MDD samples (Fig. 2B). Further GO analyses of the 113 common DEGs identified 8 enriched BP terms, including “apoptotic process” (*p* = 0.0013), “negative regulation of blood vessel endothelial cell migration” (*p* = 0.0188), and “xenobiotic metabolic process” (*p* = 0.0209); 8 CC terms, including “cytoplasm” (*p* = 0.0011), “cytosol” (*p* = 0.0090), and “cytosolic small ribosomal subunit” (*p* = 0.0235); and 8 MF terms, including “zinc ion binding” (*p* = 0.0095), “gluconokinase activity” (*p* = 0.0112), and “metalloendopeptidase activity” (*p* = 0.0326) (Fig. 2C). KEGG analysis identified 28 enriched pathways, including “transcriptional misregulation in cancer” (*p* = 0.0002), “metabolic pathways” (*p* = 0.0003), “endometrial cancer” (*p* = 0.0007), “prolactin signaling pathway” (*p* = 0.0012), and “MAPK signaling pathway” (*p* = 0.0110) (Fig. 2C).

### PPI network construction and identification of hub genes

**Fig. 3:**
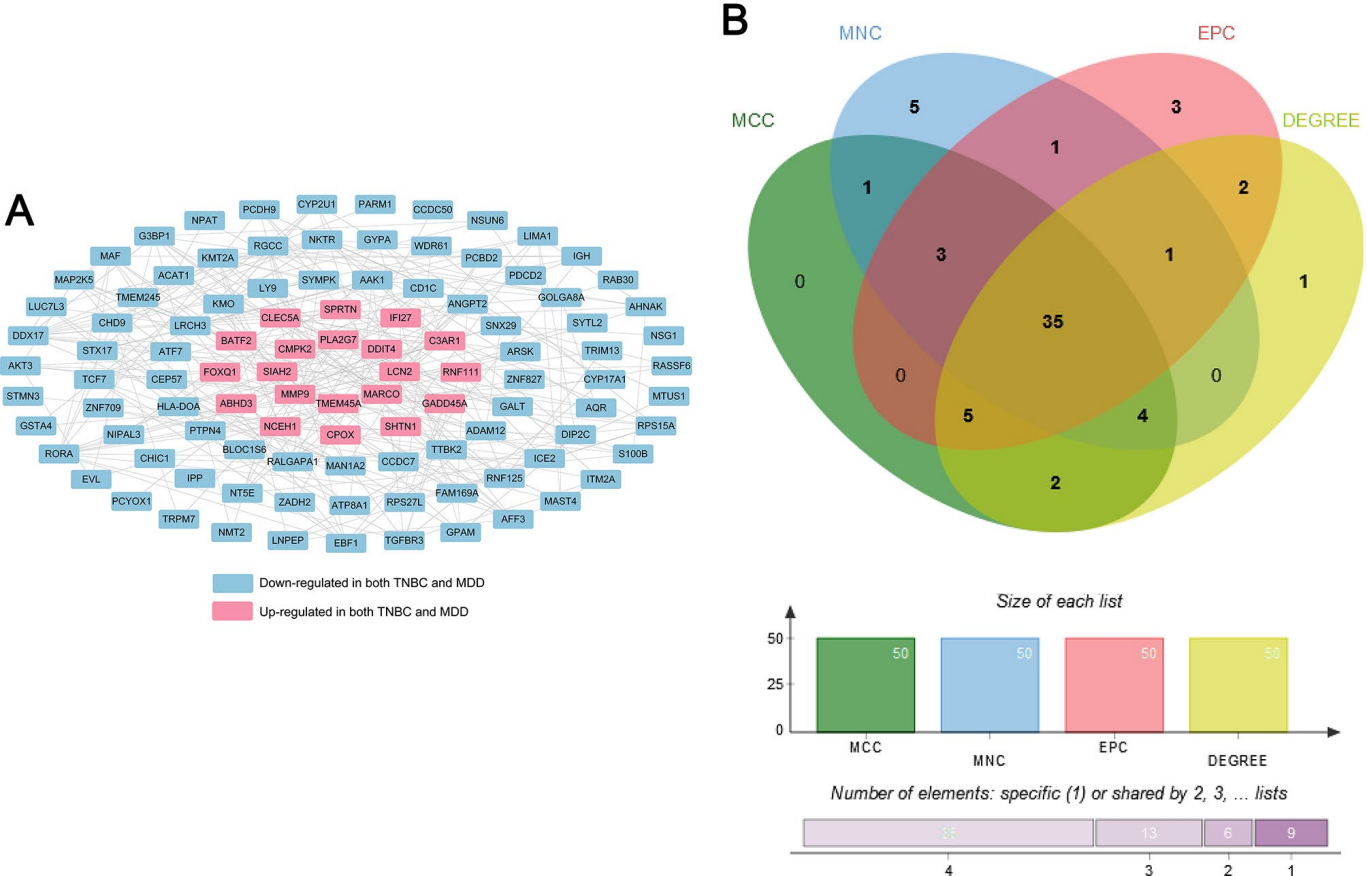
Protein-protein interaction (PPI) network construction and selection of hub genes.(**A**) PPI network of the common DEGs. (**B**) A Venn diagram showing the hub genes derived from four different methods

A total of 266 pairs of interactions were potentially related to the 113 DEGs, according to the STRING database (Fig. 3A). The intersection of results from the four different topological methods generated 35 hub genes for this network (Fig. 3B).

### WGCNA and selection of hub DEGs

**Fig. 4:**
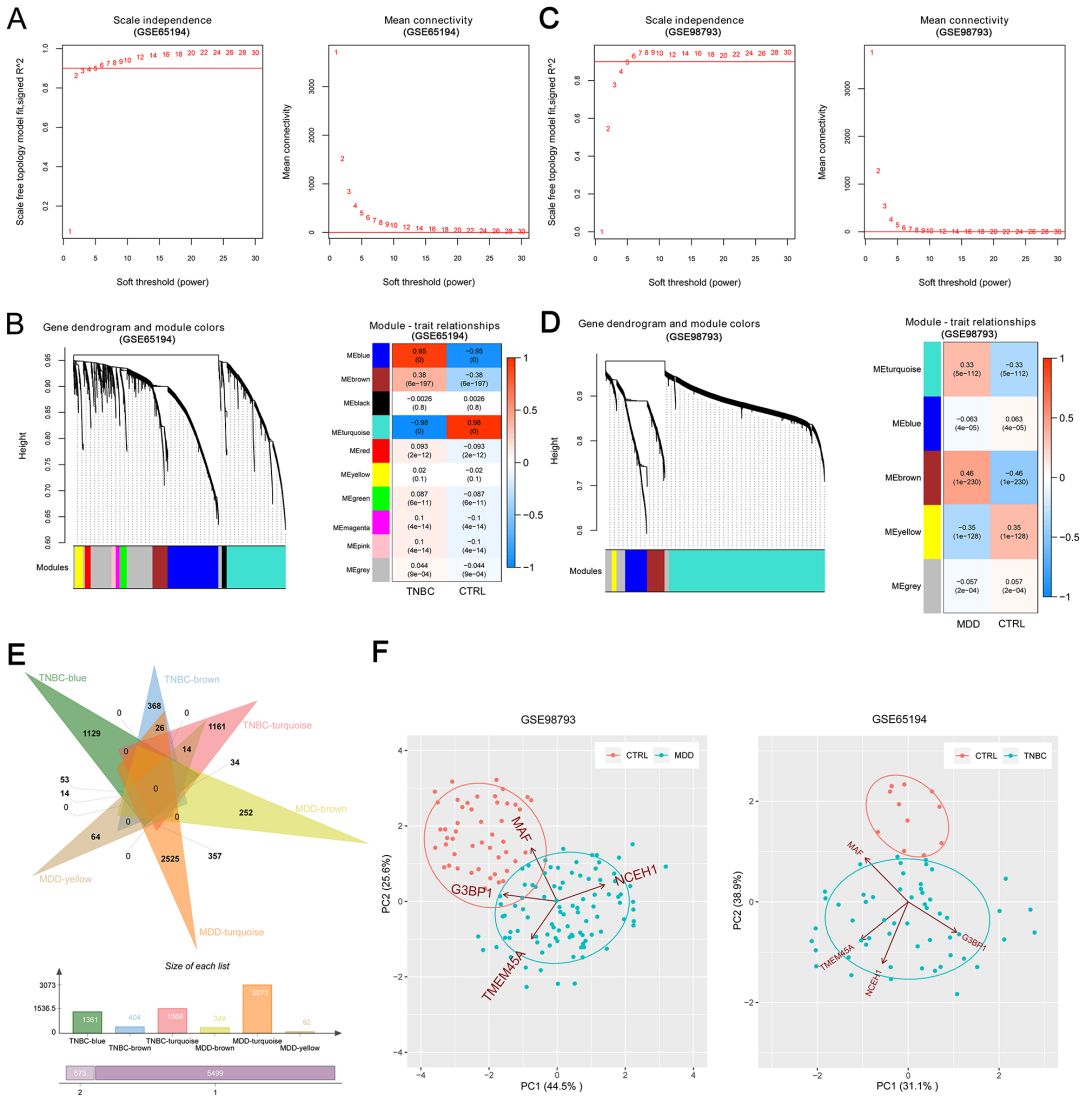
Weighted gene co-expression network analysis (WGCNA) and selection of hub DEGs.(**A**) Selection of the optimal soft threshold power in GSE65194. (**B**) A clustering dendrogram (left) and a heat map (right) showing the correlations between WGCNA modules and the disease in GSE65194. (**C**) Selection of the optimal soft threshold power in GSE98793. (**D**) A clustering dendrogram (left) and a heat map (right) showing the correlations between WGCNA modules and the disease in GSE98793. (**E**) A Venn diagram showing the intersection of DEGs from disease-associated WGCNA modules. (**F**) A principal component analysis diagram illustrating the separation of samples into distinct groups based on the expression levels of hub DEGs.

For both TNBC and MDD training sets, 6 was chosen as the optimal soft threshold power to build the scale-free weighted co-expression network, with a mean connectivity of 1 (Fig. 4A and C), qualifying it as a small-world network. After merging the modules with highly identical gene expression, ten and five modules were obtained in the co-expression networks for the TNBC and MDD training sets, respectively (Fig. 4B and D). The module-trait relationships between diseases and modules were further analyzed (Fig. 4B and D). Next, we extracted genes from the modules that were significantly positively correlated with TNBC (e.g., TNBC-blue, TNBC-brown, and TNBC-turquoise) and MDD (e.g., MDD-brown, MDD-turquoise, and MDD-yellow) and identified 673 genes that were correlated with both diseases (Fig. 4E). These 673 genes intersected with the previously identified 35 hub genes, and four genes that were present in both sets, namely *G3BP1, MAF, NCEH1* and *TMEM45A*, were chosen as hub DEGs. The principal component analysis (PCA) diagram separated the samples into distinct groups according to the expression levels of the hub DEGs (Fig. 4F).

### Validation of the expression levels of the hub DEGs

**Fig. 5:**
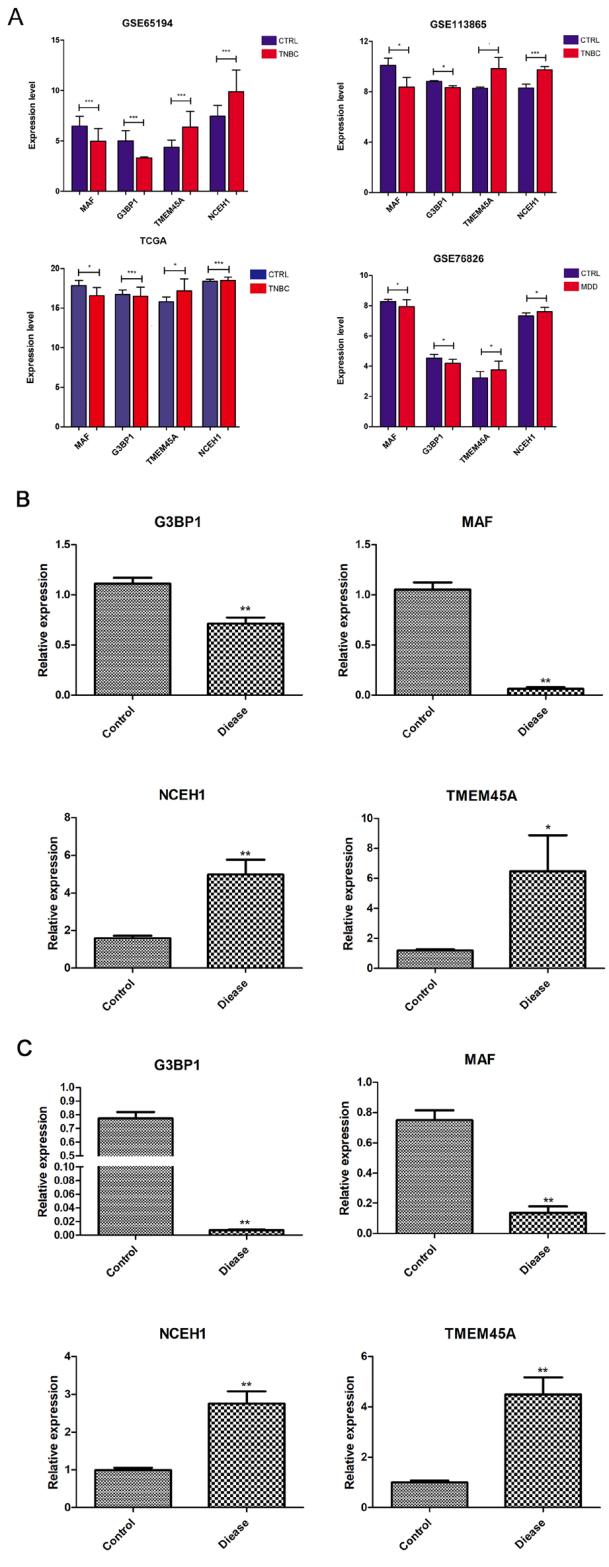
Validation and annotation of hub DEGs and their potential interactions with miRNAs.(**A**) Expression levels of hub DEGs in the training and validation sets. (**B**) The expression levels of hub DEGs in the tissue samples measured by qRT-PCR. (**C**) The expression levels of hub DEGs in the blood samples measured by quantitative real-time reverse transcription-polymerase chain reaction (qRT-PCR). * indicates *p* < 0.05, ** represents *p* < 0.01, and *** indicates *p* < 0.001

In all datasets, the expression of *G3BP1* and *MAF* was significantly downregulated in the TNBC and MDD groups compared to that in the control group, and the expression of *NCEH1* and *TMEM45A* was significantly upregulated in the TNBC and MDD groups compared to that in the control group (Fig. 5A). In addition, the same trend was observed in the blood of patients with MDD and in tissue samples from patients with BC using RT-qPCR (Fig. 5B and C). The detailed baseline characteristics of the patients and healthy subjects from whom blood and tissue samples were collected are listed in Tables 2 and 3.

**Table 2:** Baseline characteristics of the breast cancer patients

Patient ID	Gender	Age (year)	BMI (kg/m^2^)	Tumor location	Histological type	TNM stage	Tumor size	Metastatic status	Nottingham grade
RX1	Female	48	24.97	left-sided	invasive carcinoma	T1cN0	1.4*1.0	No metastasis	3
RX2	Female	55	23.11	right-sided	invasive carcinoma	T2N1a	3.5*3.0	Axillary lymph node (1/19) positive	3
RX3	Female	39	24.97	left-sided	invasive carcinoma	T2N0	3.9*3.9*3.6	No metastasis	3
RX4	Female	54	19.72	left-sided	invasive carcinoma	T2N0	3.5*3.5*2.0	No metastasis	3
RX5	Female	53	22.22	left-sided	invasive carcinoma	T2N1a	2.9*2.5	Axillary lymph node (1/17) positive	3

BMI: body weight index; TNM: tumor-node-metastasis

**Table 3:** Baseline characteristics of the major depressive disorder patients and the matched healthy individuals

Patient ID	Group	Gender	Age (year)	BMI (kg/m^2^)	Age of disease onset (year)	HAMD score
S11	patient	Female	22	21.48	20	57
S22	patient	Female	23	18.29	20	52
S33	patient	Female	22	22.76	22	51
S44	patient	Female	49	20.43	46	55
S55	patient	Female	58	22.86	36	56
D11	healthy	Female	22	22.37		
D22	healthy	Female	24	25.21		
D33	healthy	Female	20	22.07		
D44	healthy	Female	50	24.65		
D55	healthy	Female	58	22.54		

BMI: body weight index; HAMD: Hamilton Depression Rating Scale

### Identification of the disease-associated “miRNA-hub DEG” interactions and functional annotation of the hub DEGs

**Fig. 6:**
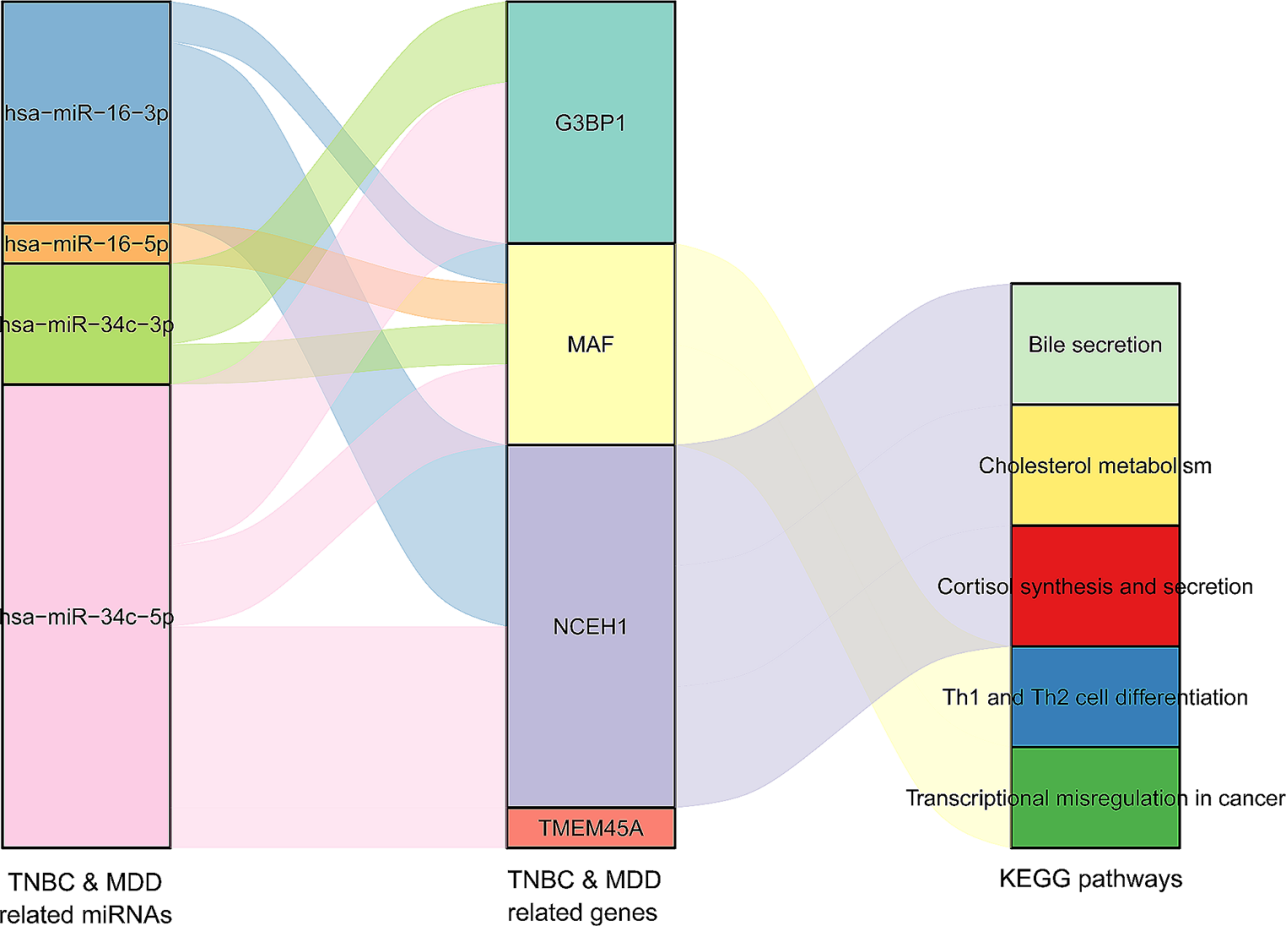
“microRNA-hub DEG” interactions and functional annotation of hub DEGs. A Sankey diagram showing the regulatory relationships between disease-associated miRNAs and hub DEGs, and the annotated Kyoto Encyclopedia of Genes and Genomes pathways of the hub DEGs.

We identified 67 TNBC-associated miRNAs and 14 MDD-associated miRNAs from the HMDD database, of which hsa-miR-34c and hsa-miR-16 were correlated with both diseases. miRNAs predicted to regulate hub DEGs were downloaded from the miRWalk database, and 17 miRNA-target pairs implicated hsa-miR-34c and hsa-miR-16 (Fig. 6). The annotated KEGG pathways of the hub DEGs were “cholesterol metabolism,” “cortisol synthesis and secretion,” “bile secretion,” “Th1 and Th2 cell differentiation,” and “transcriptional misregulation in cancer” (Fig. 6).

## Discussion

In this study, we identified 20 genes that were upregulated in both TNBC and MDD and 93 genes that were downregulated in both diseases. From these 113 genes, PPI network construction and WGCNA led to the identification of four hub DEGs that may play the most critical roles in the pathogenesis of both diseases, namely *G3BP1, MAF, NCEH1* and *TMEM45A*. Subsequently, their expression levels in public datasets as well as in blood and breast tissue samples were validated. Hsa-miR-34c and hsa-miR-16 were predicted to regulate the expression of hub DEGs using the HMDD database.

GO analyses uncovered that the common DEGs of both diseases were functionally related to biological pathways such as “apoptotic process,” “negative regulation of blood vessel endothelial cell migration,” and “xenobiotic metabolic process.” Moreover, they were enriched in molecular activities including “zinc ion binding,” “gluconokinase activity,” and “metalloendopeptidase activity.” Genetic variants of xenobiotic metabolism genes have been associated with the risk of breast cancer [21]. Studies on neurodegenerative disorders, including depression, have provided evidence that xenobiotic-metabolizing enzymes have important functions in brain physiology [22]. *NCEH1*, a common DEG in GO-BP, encodes a multifunctional enzyme that hydrolyzes the amide and ester bonds of many xenobiotic chemicals [23]. In addition, gluconokinases such as IDNK have been reported to promote cancer cell proliferation and inhibit apoptosis [24]. Furthermore, a number of studies on MDD have implicated glucose metabolic dysfunction in the pathophysiology of MDD, although the underlying molecular mechanisms remain elusive [25]. Common DEGs in this GO-MF term included *IDNK*. Moreover, certain matrix metalloproteinases belonging to the metalloendopeptidase family, such as MMP-9, have been found to contribute significantly to the pathophysiology of depression [26] and have also been identified as therapeutic targets for metastatic breast cancer [27]. In the present study, *MMP-9* was found to exhibit “metalloendopeptidase activity.” In addition, KEGG analysis revealed that the common DEGs were enriched in 28 pathways including “transcriptional misregulation in cancer,” “metabolic pathways,” “endometrial cancer,” “prolactin signaling pathway,” and “MAPK signaling pathway.” Prolactin was observed to promote bone metastasis in breast cancer patients, possibly by stimulating lytic osteoclast formation [28], and data from an animal model of MDD also supported the pathological role of prolactin in MDD [29]. MAPK signaling is one of the aberrantly activated oncogenic pathways in breast cancer [30]. This pathway was also implicated in the activation of the pro-inflammatory transcription factor NF-kappaB, potentially contributing to the pathogenesis of MDD in which inflammation is a key pathological element [31]. In summary, the results from the functional enrichment analyses of our study are in line with previous findings and suggest that the above-mentioned pathways and biological activities may play important roles in the pathology of both TNBC and MDD.

From further PPI network construction and WGCNA we obtained four hub DEGs (e.g., *G3BP1, MAF, NCEH1* and *TMEM45A*), and using the HMDD database we identified that hsa-miR-34c and hsa-miR-16 possibly regulate the hub DEGs. Of the four hub DEGs, *NCEH1* (which encodes a versatile enzyme involved in diverse metabolic processes) attracted our attention. In addition to its role in xenobiotic metabolism, this enzyme is critical for providing cholesterol for the synthesis of bile acids (BAs) because of its ability to hydrolyze cholesterol esters [32]. Abnormal serum levels or altered composition of BAs have been implicated in the pathogenesis of both BC and MDD [33, 34]. Our results showed that *NCEH1* expression was significantly elevated in both TNBC and MDD, indicating that it might play a pathogenic role in these two diseases. It is important to know how its expression levels affect BA synthesis and secretion and whether altered BA levels result in the development of TNBC and MDD. It is also possible that the major pathogenic effects of *NCEH1* overexpression are mediated by other molecules and pathways, which warrants further exploration. Furthermore, hsa-miR-34c and hsa-miR-16 were predicted to regulate the expression *NCEH1* in the HMDD database, and ample evidence has demonstrated the important roles of these two miRNAs in TNBC and MDD. Low plasma levels of hsa-miR-34c have been reported to be associated with poor prognosis in TNBC [35], and hsa-miR-34c has also been found to suppress TNBC invasiveness and epithelial-mesenchymal transition [36]. Hsa-miR-16 has been shown to inhibit the proliferation, invasion, and migration of TNBC cells [37, 38] and has diagnostic value for TNBC [39]. Additionally, significant negative correlations have been identified between hsa-miR-34c levels and MDD symptoms [40], and both blood and cerebrospinal fluid levels of hsa-miR-16 been found to be significantly downregulated in patients [41–43]. Neither of these miRNAs has been experimentally validated to regulate *NCEH1* so far. Thus, future assays are needed to verify their regulatory relationships in vivo.

Our study had some limitations. First, the clinical information available in public databases is limited and not all datasets are of substantial size, which could introduce bias into our findings. Secondly, the sample size used in our experiments was relatively small, necessitating further validation through larger-scale studies. Lastly, there is insufficient evidence to conclusively designate *G3BP1*, *MAF*, *NCEH1*, and *TMEM45A* as potential therapeutic targets for TNBC and MDD. This hypothesis requires future clinical trials for verification.

In conclusion, *G3BP1, MAF, NCEH1* and *TMEM45A* may be regulated by hsa-miR-34c and hsa-miR-16 may play critical roles in the pathogenesis of TNBC and MDD. Xenobiotic metabolism, gluconokinase, matrix metalloproteinase, prolactin and MAPK signaling pathways, and bile secretion may underlie the development of these two diseases. These findings provide novel insights for future research on biomarkers and therapeutic targets of both diseases.

## Data Availability

The data and materials that support the findings of this study are available on request from the corresponding author.
